# Supplementary material for: Comprehensive characterization of RAS mutations in colon and rectal cancers in old and young patients
Source: Nat Commun. 2019 Aug 19;10:3722. doi: 10.1038/s41467-019-11530-0 (PMC6700103; doi:10.1038/s41467-019-11530-0)
Supplement: Supplementary file 1 — Supplementary Information [file 41467_2019_11530_MOESM1_ESM.pdf]

Comprehensive characterization of *RAS* mutations in colon and rectal cancers in old and young patients

Supplementary Information

Serebriiskii et al.

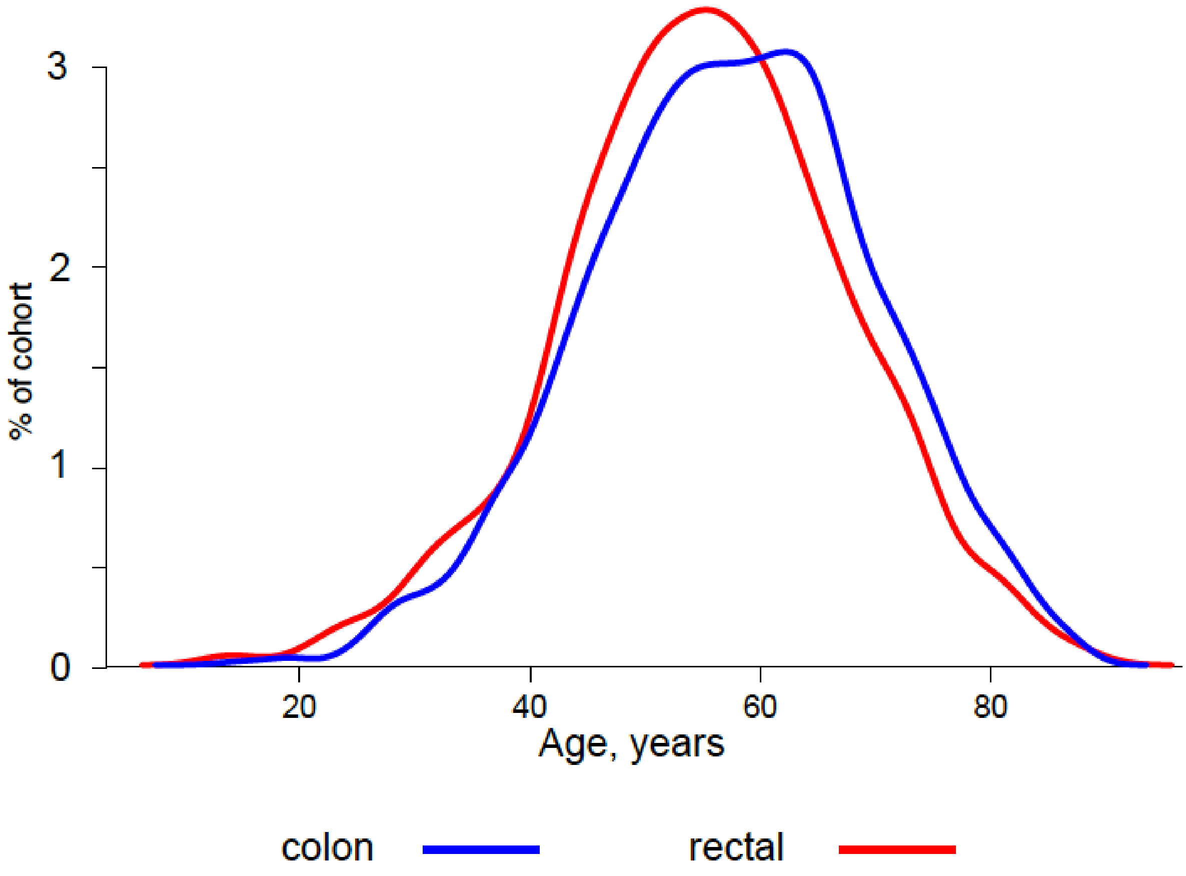

Supplementary Figure 1. Age distribution of colon and rectal patients in this study. The age distribution of individuals in this study reflects slightly earlier onset of rectal cancer compared to colon cancer.

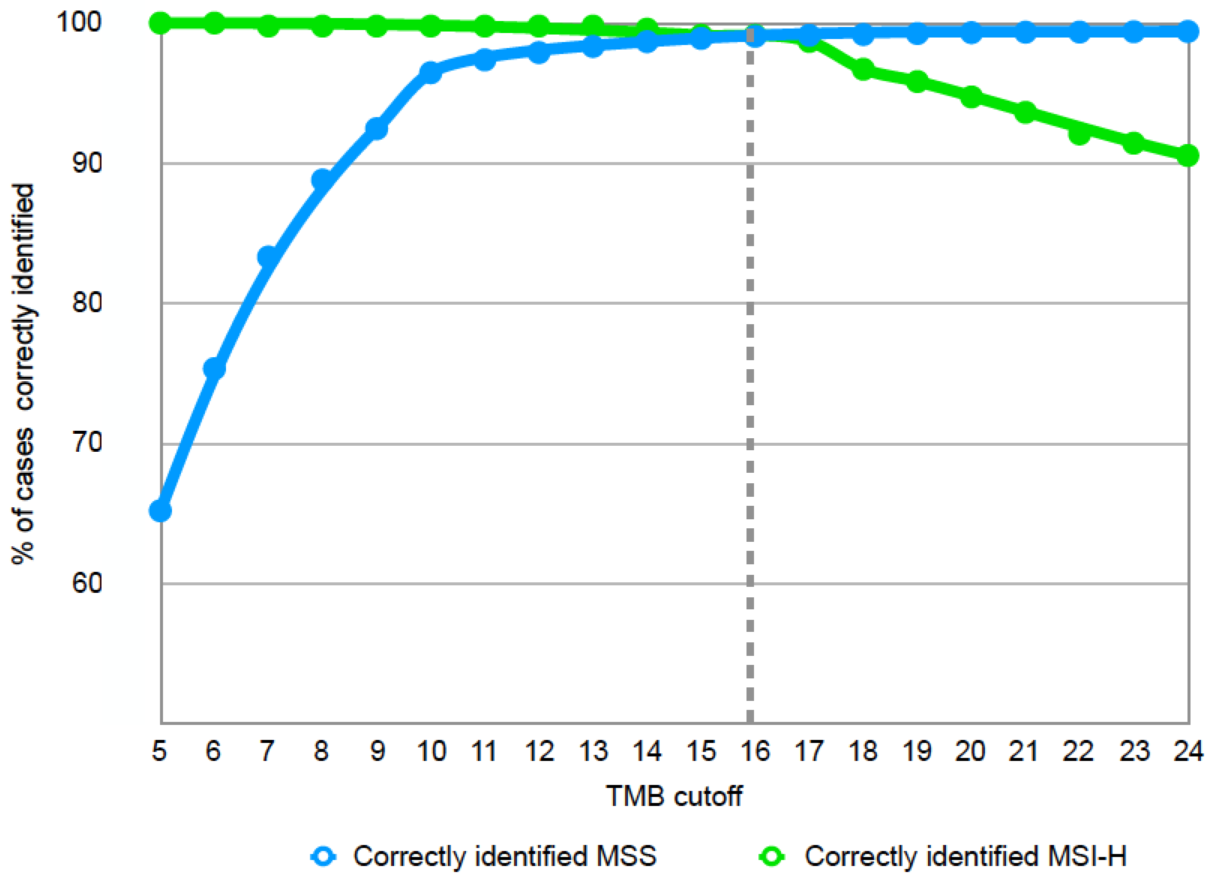

Supplementary Figure 2. Selection of MSI-H/MSS status based on Tumor Mutation Burden (TMB). Graph indicates % cases of MSH-H (red line) versus MSS (blue line) status identified in training set based on presence of indicated number of mutations in specimen. A cutpoint of 16 mutations was used to segregate the groups as MT-H or MT-L.

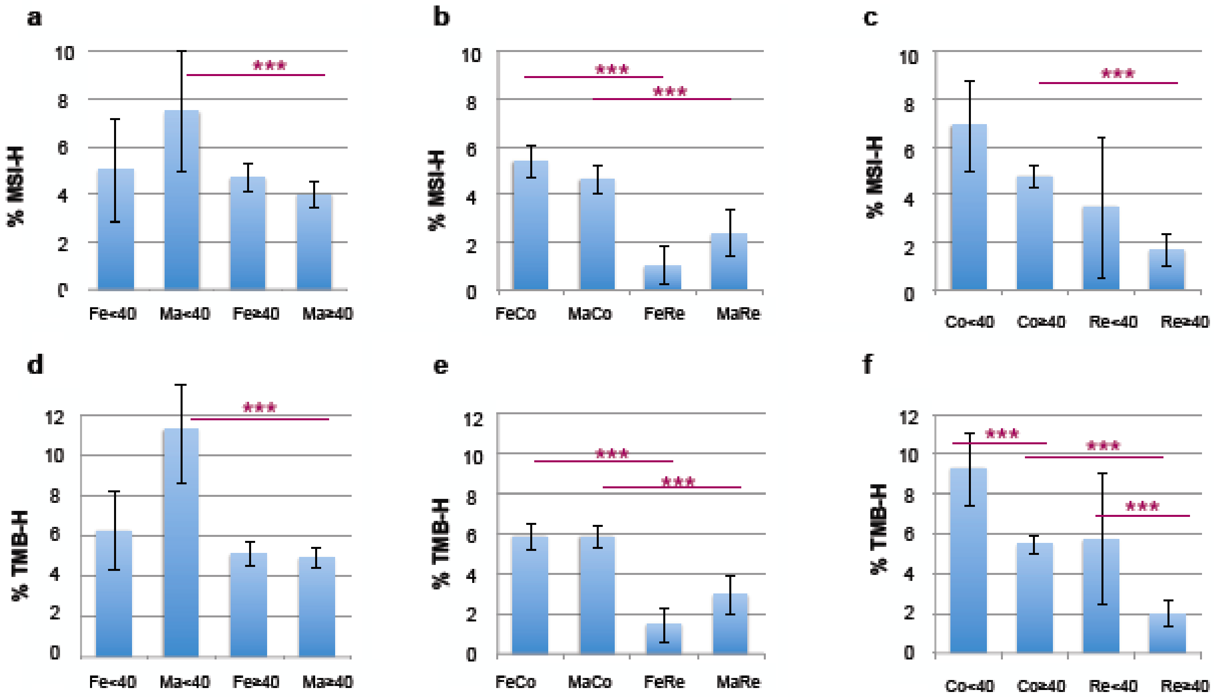

Supplementary Figure 3. Overall profile of dataset, MSI-H and TMB. Frequency of MSI-H (**a-c**) or TMB-H (**d-f**) tumors based on parameters of gender (Fe, female or Ma, male) as a factor of age (<40 or ≥40) (**a,d**), or primary tumor site (Co, colon or Re, rectum) (**b, e**); or in combined genders, based on age and tumor site (**c, f**). Error bars, 95% confidence intervals. Statistical significance is denoted by ***, $p<0.005$.

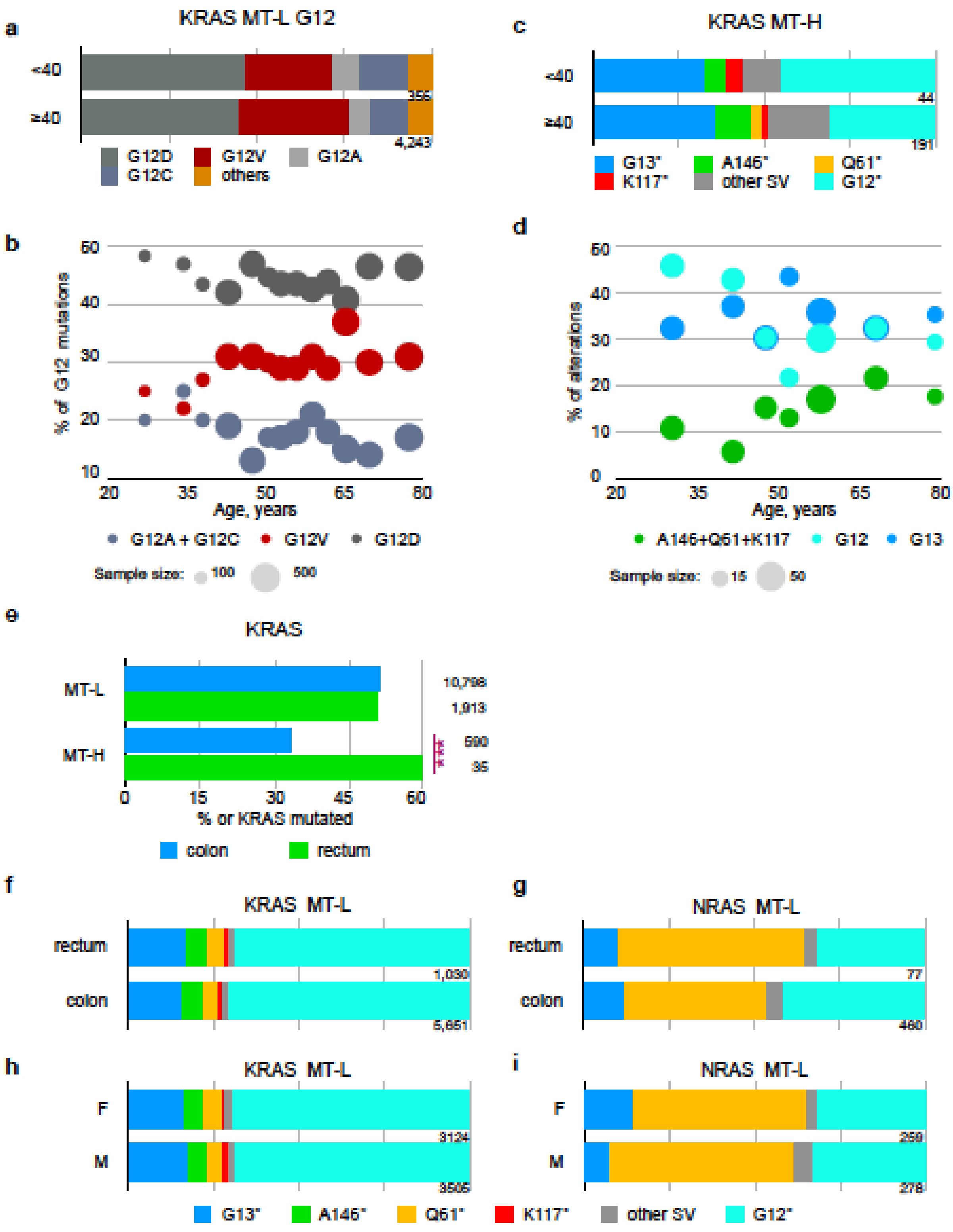

Supplementary Figure 4. Additional comparisons of mutational spectrum in *RAS* genes. a, b. Frequency of specific missense mutations *KRAS* G12 in MT-L CRC patients dichotomized by age

age (<40 or ≥40) (**a**) or with age considered as a continuous variable (**b**). **c, d**. Frequency of mutations in the indicated codons in *KRAS* in MT-H patients dichotomized by age (<40 or ≥40) (**c**) or with age considered as a continuous variable (**d**). For pale blue circles outlined in dark blue, G12 and G13 frequency is equivalent. **e**. Frequency of *KRAS* missense mutations in MT-L and MT-H tumors of the colon and rectum. **f**, **g**. Frequency of mutations in the indicated codons in *KRAS* (**f**) or *NRAS* (**g**) MT-L CRC based on tumor subsite (colon versus rectum). **h**, **i**. Frequency of mutations in the indicated codons in *KRAS* (**h**) or *NRAS* (**i**) MT-L CRC based on gender.
