# Supplementary material for: Comprehensive characterization of RAS mutations in colon and rectal cancers in old and young patients
Source: Nat Commun. 2019 Aug 19;10:3722. doi: 10.1038/s41467-019-11530-0 (PMC6700103; doi:10.1038/s41467-019-11530-0)
Supplement: Supplementary file 3 — Description of Additional Supplementary Files [file 41467_2019_11530_MOESM3_ESM.pdf]

Description of Additional Supplementary Information

File Name: Supplementary Data 1.

Description: Summary of protein and corresponding nucleotide changes resultingin KRAS and NRAS mutations. All recurrent nucleotide changes (found in the analyzed set at least four times), were retrieved. 38 mutations, with a combined total of 7435 occurrences, represent 97.3% of all structural variations in KRAS and/or NRAS genes in the dataset; information for the remaining 2.7% of rare changes ae available on request. Four occurrences of different nucleotide changes resulting in the same amino acid changes, are highlighted.
